# Elevated autoantibody content in rheumatoid arthritis synovia with lymphoid aggregates and the effect of rituximab

**DOI:** 10.1186/ar2497

**Published:** 2008-09-01

**Authors:** Sanna Rosengren, Nathan Wei, Kenneth C Kalunian, Nathan J Zvaifler, Arthur Kavanaugh, David L Boyle

**Affiliations:** 1 Division of Rheumatology, Allergy and Immunology, University of California at San Diego School of Medicine, 9500 Gilman Drive, La Jolla, CA 92093, USA; 2 Arthritis and Osteoporosis Center of Maryland, 71 Thomas Johnson Drive, Frederick, MD 21702, USA

## Abstract

**Introduction:**

The purpose of this study was to quantitatively evaluate the contribution of synovial lymphoid aggregates to autoantibody (rheumatoid factor [RF] and anti-cyclic citrullinated peptide [anti-CCP]) and total immunoglobulin (IgG and IgM) production in rheumatoid arthritis (RA) patients and the effect thereon of the B-cell-depleting antibody, rituximab, in the ARISE (Assessment of Rituximab's Immunomodulatory Synovial Effects) trial.

**Methods:**

Autoantibodies as well as total IgM and IgG were quantified by enzyme-linked immunosorbent assay in extracts of synovial tissues and matched serum from patients with RA or osteoarthritis (OA). Synovial biopsies and serum were obtained at baseline and 8 weeks following rituximab therapy in 14 RA patients. A synovial/serum index (SSI) was calculated as the ratio of synovial to serum antibody/albumin, with values above 1 representing synovial enrichment. Lymphoid aggregates were evaluated histologically.

**Results:**

Anti-CCP IgG, but not RF-IgM, was significantly enriched in RA synovia compared with serum. Total IgM and IgG were also enriched in RA, but not in OA. SSI correlated significantly with mRNA content for both IgM and IgG, demonstrating that it reflected synovial immunoglobulin production. RA synovia with lymphocyte aggregates contained significantly elevated RF-IgM and anti-CCP IgG compared with tissues with diffuse lymphoid infiltration. Rituximab treatment did not affect synovial autoantibody or total immunoglobulin SSI overall. However, in aggregate-containing tissues, rituximab significantly reduced total IgM and IgG SSI as well as IgM and IgG1 mRNA. Surprisingly, RF-IgM and anti-CCP IgG SSIs were unchanged by rituximab in aggregate-containing synovia.

**Conclusions:**

Combined with earlier observations that synovial lymphoid aggregates are unaltered by rituximab treatment, these data suggest that lymphoid aggregates may provide a protective niche for autoantibody-producing cells.

**Trial Registration:**

The ARISE trial is registered at ClinicalTrials.gov as number NCT00147966.

## Introduction

Rheumatoid arthritis (RA) is associated with the presence of certain circulating autoantibodies, such as rheumatoid factors (RFs) and anti-cyclic citrullinated peptide (anti-CCP) [1]. The latter has received recent attention because elevated levels can precede development of joint symptoms and because it acts synergistically with the shared HLA-DR epitope to enhance the risk of developing RA [2]. A contribution of B cells and their products to the pathogenesis of RA is supported by the clinical success of rituximab, a B-cell-depleting antibody targeting CD20. Whereas long-lived plasma cells are unaffected by rituximab, circulating B cells are nearly completely depleted [3,4] and modest, albeit significant, decreases in circulating RF and anti-CCP antibodies are observed [5]. The effect of rituximab on the rheumatoid synovium is just beginning to be characterized. Recently, we [6] and others [7] reported that, following rituximab treatment, synovial B cells are depleted less effectively, and more variably, than their circulating counterparts. In the subset of patients with synovial lymphoid aggregates, rituximab treatment did not alter the number or size of these aggregates [7]. Because such aggregates are associated with elevated synovial immunoglobulin synthesis, as determined by mRNA levels for IgG constant regions [8], and perhaps also autoantibody synthesis, we sought to determine the effect of rituximab treatment on synovial autoantibody production.

The local synthesis of immunoglobulins and autoantibodies by rheumatoid synovium is well appreciated but its contribution to the circulating pool is poorly understood. Explants of rheumatoid synovial tissue are capable of synthesizing immunoglobulins [9,10], RF [9,10], and anti-CCP IgG [11]. Similarly, dispersed cells from rheumatoid synovia synthesize immunoglobulins [12,13] and RF [13-15], and synovial fluid-derived mononuclear cells secrete anti-CCP antibodies [16]. Although these techniques are valuable for the understanding of the contribution of local antibody synthesis to the pathogenesis of RA, their applicability in interventional biopsy-based clinical trials is limited. Synovial tissues obtained by arthroscopy or needle biopsy typically do not yield enough tissue to recover a sufficient amount of dispersed cells, and the viability of synovial biopsies for explant cultures might be compromised when samples have to be transported from clinical sites to the laboratory. With this in mind, we developed and validated a novel set of techniques that can be used on frozen specimens for the measurement of autoantibodies and immunoglobulins in paired synovial biopsies and sera obtained prior to, and following, an intervention. These methods were used to evaluate the effect of rituximab treatment on synovial autoantibody and immunoglobulin production and the role of lymphoid architecture on this effect.

## Materials and methods

### Patients

Patients with RA or osteoarthritis (OA) were included after informed consent was obtained under approval from the University of California-San Diego Institutional Review Board. A subset of patients who were part of the ARISE (Assessment of Rituximab's Immunomodulatory Synovial Effects) clinical trial, recently described in detail [6], received rituximab at a dose of 1 g given intravenously over the span of 4 to 5 hours on day 0 and again on day 14. The same joint was biopsied prior to and 8 weeks following treatment.

### Synovial tissue

Synovial tissue was collected at the time of joint replacement surgery (knees or hips from all OA patients and the majority of RA patients; other anatomical sites included three wrists, one shoulder, one elbow, and one metacarpophalangeal). The tissue was immediately placed on ice and transported to the laboratory, and synovial tissue fragments (size 1 to 2 mm^2^) were excised using a fine scalpel and snap-frozen in liquid nitrogen in sets of six or were embedded in cryosectioning medium. For ARISE patients, synovial tissue biopsies were obtained under conscious sedation anesthesia from knees or wrists using arthroscopically guided Automated Motorized Shaver technology, a method that rapidly yields greater than 50 synovial tissue fragments rich in synovial lining. Aliquots of the resulting synovial fragments were immediately snap-frozen or embedded. In some cases, a paired serum or plasma sample was obtained at the time of surgery or biopsy. All samples were stored at -80°C until analysis. The presence or absence of lymphoid aggregates was scored on hematoxylin/eosin-stained cryosections. Tissues displaying grade 2 or 3 aggregates [17] were considered positive for the presence of lymphoid aggregates.

### Autoantibody and immunoglobulin enzyme-linked immunosorbent assays

Frozen synovial fragments were weighed and immediately placed in chilled 1-mL Kontes-Duall tissue grinders, and ice-cold extracting buffer consisting of 1% Brij-35 detergent (Sigma-Aldrich, St. Louis, MO, USA) in phosphate-buffered saline with protease inhibitor cocktail (Complete Mini; Roche Applied Science, Indianapolis, IN, USA) was added at 50 μL per 10 mg of tissue. The mixture was ground by hand on ice until only fibrous white insoluble material remained. After incubation of the mixture for at least 10 minutes on ice, it was transferred to a microcentrifuge tube and centrifuged 10 minutes at 20,000 *g *and 4°C. The resulting supernatant was aliquotted and stored at -80°C for later analysis. Total protein content in extracts diluted 1:10 in distilled water was determined using DC Protein Assay reagents (Bio-Rad Laboratories, Hercules, CA, USA). Colorimetric enzyme-linked immunosorbent assay kits were used to detect RF of the IgM subtype (RF-IgM) (ALPCO Diagnostics, Salem, NH, USA), anti-CCP IgG (INOVA Diagnostics, Inc., San Diego, CA, USA), anti-tetanus IgG (ImmunoBiological Laboratories, Minneapolis, MN, USA), total IgM and total IgG, and albumin (all from Bethyl Laboratories, Montgomery, TX, USA) in synovial extracts and serum or plasma samples diluted to yield absorbance values in the linear range of the kit. Standard curves were constructed by regression line fitting on log(absorbance) versus log(concentration). In preliminary experiments, recovery of spiked standards in synovial extracts was assessed as described earlier [18] and found to be better than 80% in all cases. Analysis was performed on pools of greater than six synovial tissue fragments in order to minimize the effects of synovial heterogeneity [18]. For a given autoantibody or immunoglobulin analyte, synovial/serum index (SSI) was defined as the ratio of synovial analyte/synovial albumin divided by the ratio of serum analyte/serum albumin.

### Heavy chain constant region quantitative real-time polymerase chain reaction

Messenger RNA for heavy chain constant regions for IgM and IgG1 was quantified by real-time TaqMan quantitative real-time polymerase chain reaction (qPCR) using cDNA with glyceraldehyde-3-phosphate dehydrogenase (GAPDH) used as a housekeeper. We have previously established that data derived from sets of six or more synovial tissue fragments minimize sampling error [19] and pools of greater than six fragments were used for qPCR analysis. Resulting threshold cycle data were normalized to standard curves constructed from cDNA from RAMOS (IgM), ARH-77 (IgG1), and human PBMC (GAPDH) [19], yielding cell equivalents. The ratio between the specific cytokine and GAPDH cell equivalents (relative expression units, REU) is reported.

### Statistical analysis

Data are expressed as median and quartile and were analyzed by Wilcoxon rank sum test for comparing two unpaired groups. The Wilcoxon sign rank test was used to test locations of nonparametric populations. The effect by rituximab on synovial REU, autoantibody, and immunoglobulin levels is expressed as the geometric mean ± 95% confidence interval (CI) of percentage change pre- to post-treatment [20].

## Results

### Detection of autoantibodies in synovial tissue extracts

**Figure 1:**
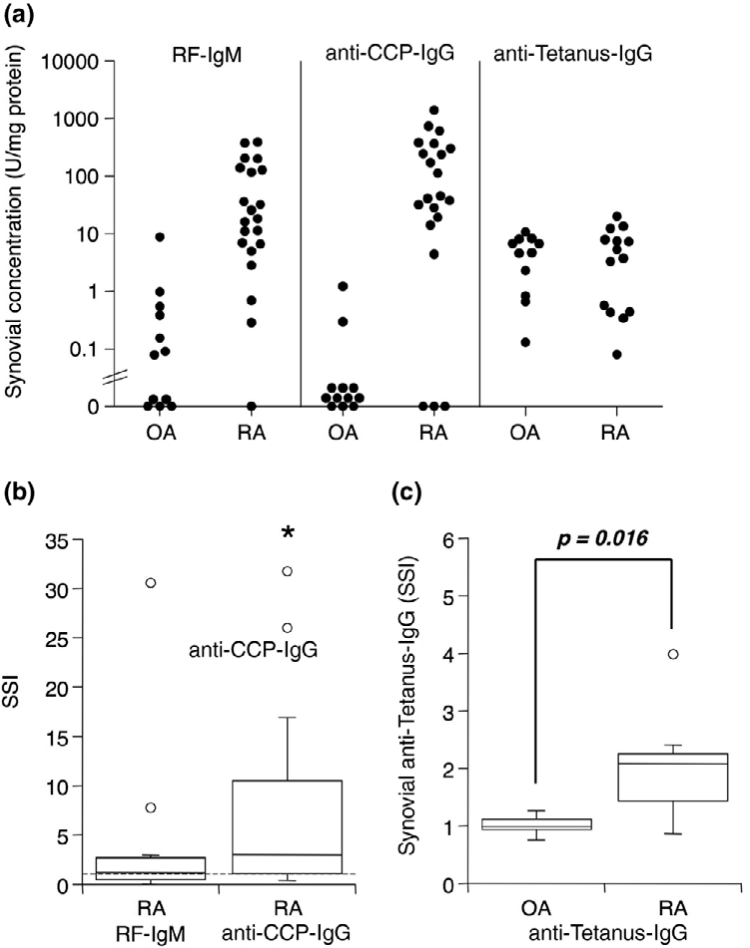
Detection and enrichment of autoantibodies in rheumatoid arthritis (RA) synovial extracts. Tissues were obtained by arthroplasty or arthroscopic shaver biopsy, and serum and synovial extracts were analyzed by enzyme-linked immunosorbent assay for antibodies of interest and albumin. **(a) **Individual levels of RF-IgM, anti-CCP IgG, and anti-tetanus IgG in extracts from osteoarthritis (OA) (n = 12, autoantibodies; n = 11, anti-tetanus) and RA (n = 21, autoantibodies; n = 14, anti-tetanus) synovial tissue, normalized to total protein concentration. Limits of detection are 4 (RF-IgM), 10 (anti-CCP IgG), and 0.3 (anti-tetanus IgG). Serum-normalized levels of **(b) **RF-IgM and anti-CCP IgG in RA synovial extracts (n = 11) and **(c) **anti-tetanus IgG in OA (n = 6) and RA (n = 9) synovial extracts. In the box (interquartile range, IQR) and whisker (maximum and minimum) plots, the horizontal line inside the box denotes median and the unfilled circles denote outliers outside IQR ± 1.5 × IQR. The asterisk denotes *P *= 0.019 by Wilcoxon sign rank test to 1 (no enrichment) for anti-CCP IgG (a), and the indicated *P *value was determined by Wilcoxon rank sum test between OA and RA for anti-tetanus IgG (b). The value for RF-IgM was not significantly above 1 (*P *= 0.32). anti-CCP, anti-cyclic citrullinated peptide; RF-IgM, rheumatoid factor of the IgM subtype; SSI, synovial/serum index.

To determine whether autoantibodies were detectable in synovia, RF-IgM and anti-CCP IgG were measured in extracts from 12 OA and 21 RA synovial tissues. Both RF-IgM and anti-CCP IgG were detectable in the majority of RA synovial extracts (17 of 21 in both cases) (Figure 1a). In contrast, OA synovial extracts were largely devoid of autoantibodies. RF-IgM was detectable in only 1 of 12 OA synovial extracts, and none contained detectable anti-CCP IgG (Figure 1a). A disease-irrelevant antibody, anti-tetanus IgG, was detectable in both RA and OA extracts at similar ranges (Figure 1a), suggesting that there were no intrinsic differences in antibody detectability between the two types of synovia. The large variability observed in autoantibody concentrations in RA extracts might be explained by varying tissue serum content and/or varying autoantibody concentrations in serum. To correct for these variables, paired synovia and sera were obtained from 11 RA and 6 OA patients, and albumin levels determined alongside antibodies to allow calculation of SSI, as described in Materials and methods. By definition, an SSI value above 1 indicates synovial enrichment. As shown in Figure 1b, anti-CCP IgG was significantly enriched in RA synovial extracts compared with serum, whereas the RF-IgM SSI was not significantly different than 1. None of the 6 OA patients was positive for either autoantibody in serum or synovial extracts, so an OA SSI could not be calculated. However, interestingly, when the specific activities for anti-tetanus IgG in RA and OA synovial extracts were compared, levels in RA extracts were significantly higher than those in OA (Figure 1c), suggesting enrichment of this disease-irrelevant antibody in RA synovia as well.

### Enrichment of total immunoglobulin in rheumatoid arthritis synovial tissue

**Figure 2:**
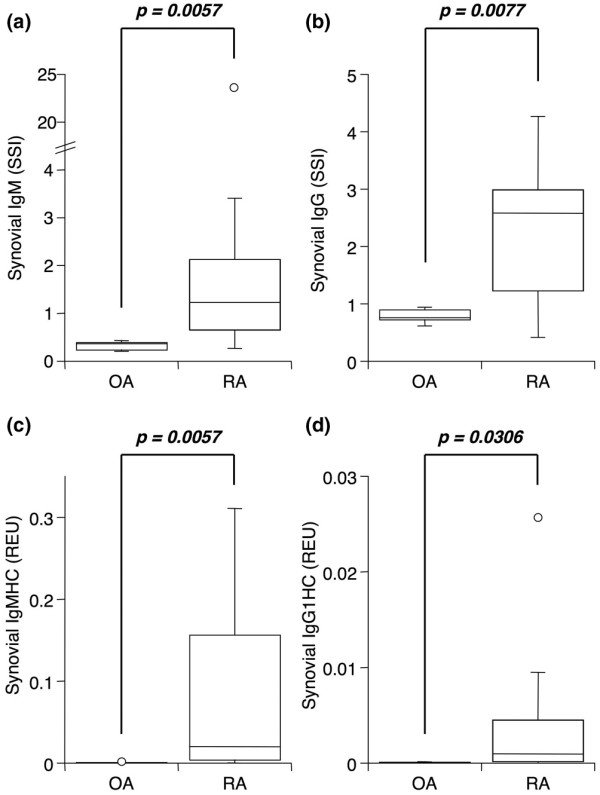
Significant enrichment of total IgM and IgG in rheumatoid arthritis (RA) synovia. Serum-normalized levels of **(a) **total IgM and **(b) **total IgG in extracts from osteoarthritis (OA) (n = 6) and RA (n = 11) synovial tissue obtained by arthroplasty or arthroscopic shaver biopsy and measured by enzyme-linked immunosorbent assay for antibodies of interest and albumin. GAPDH-normalized message for IgM **(c) **and IgG1 **(d) **heavy constant region as determined by quantitative real-time polymerase chain reaction in the same synovia as in (a) and (b). See Figure 1 legend for box plot definitions. Indicated *P *values were determined by Wilcoxon rank sum test. GAPDH, glyceraldehyde-3-phosphate dehydrogenase; REU, relative expression units; SSI, synovial/serum index.

**Figure 3:**
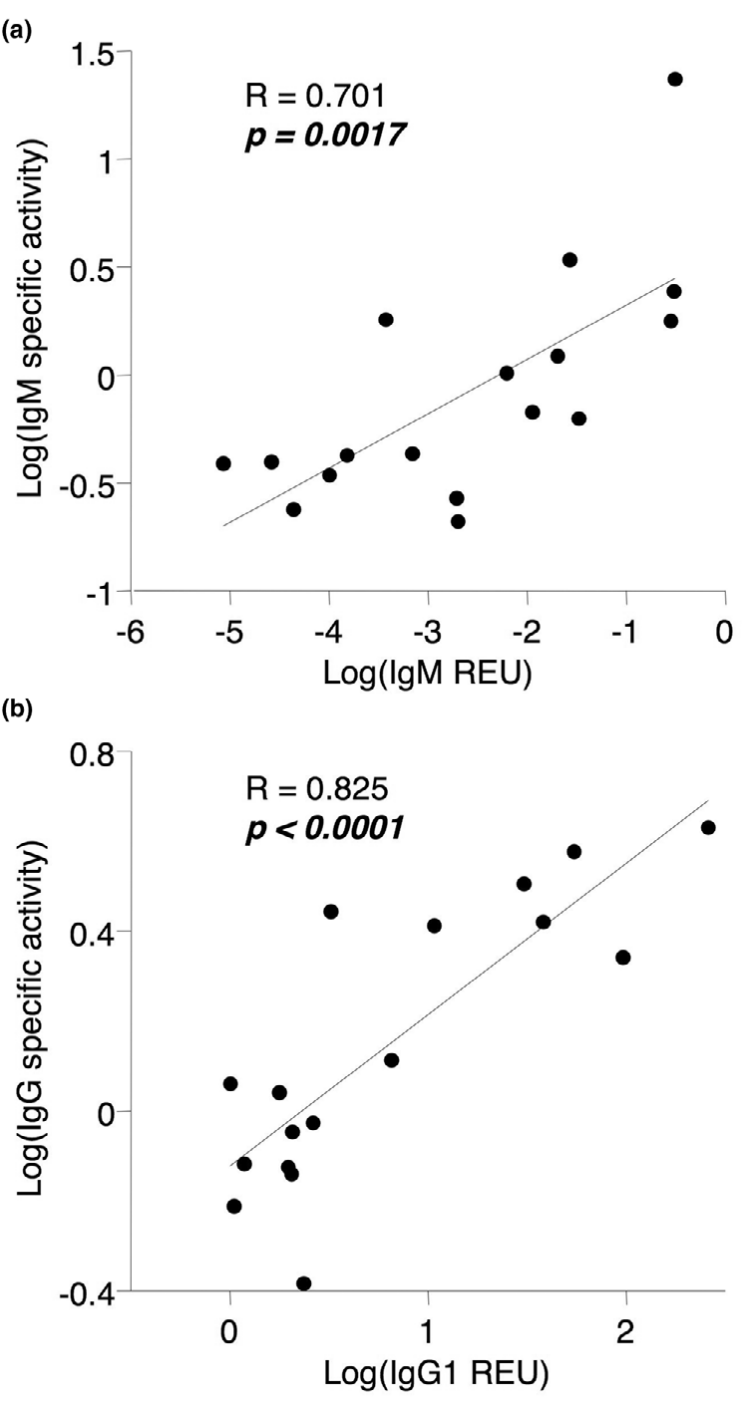
Correlation between synovial/serum index (SSI) and mRNA for total IgM and IgG. Correlation plots for SSI versus GAPDH-normalized mRNA levels for total IgM **(a) **and total IgG **(b) **in rheumatoid arthritis (RA) and osteoarthritis (OA) synovia. Data are from Figure 2. Correlation coefficients (*R*) and *P *values were determined by Spearman rank correlation. GAPDH, glyceraldehyde-3-phosphate dehydrogenase; REU, relative expression units.

Total IgM and IgG were quantified in serum and synovial extracts and specific activities calculated. Both subclasses were elevated in RA extracts compared with those in OA (Figure 2a,b). An SSI above 1 was noted in 7/11 RA and 0/11 OA samples for IgM and in 10/11 RA and 0/11 OA samples for IgG. This could indicate either enhanced local production or preferential localization to the synovium after synthesis elsewhere. To distinguish between these two possibilities, qPCR was used to determine mRNA levels for constant regions of IgM and IgG1 heavy chains in RA and OA synovia. As seen in Figures 2c and 2d, both were significantly higher in RA tissues. Notably, significant correlations were observed between mRNA levels and SSI for both IgM (*R *= 0.701; *P *= 0.0017; Figure 3a) and IgG (*R *= 0.825; *P *< 0.0001; Figure 3b), indicating that local synthesis contributes to synovial immunoglobulin enrichment.

### Autoantibodies and immunoglobulins in rheumatoid arthritis synovia with lymphoid aggregates

**Figure 4:**
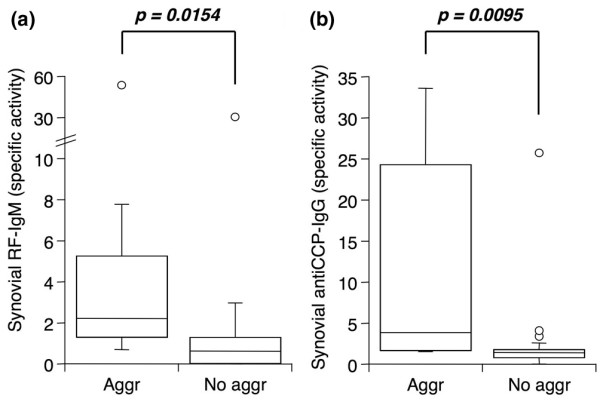
The presence of lymphoid aggregates in rheumatoid arthritis (RA) synovia is associated with elevated synovial autoantibody levels. Serum-normalized levels of **(a) **RF-IgM and **(b) **anti-CCP IgG in extracts from RA synovia with (Aggr, n = 8) or without (No aggr, n = 17) lymphoid aggregates. Tissues were obtained by arthroplasty or arthroscopic shaver biopsy, and extracts were analyzed by enzyme-linked immunosorbent assay for antibodies of interest and albumin. See Figure 1 legend for box plot definitions. Indicated *P *values were determined by Wilcoxon rank sum test. anti-CCP, anti-cyclic citrullinated peptide; RF-IgM, rheumatoid factor of the IgM subtype.

**Figure 5:**
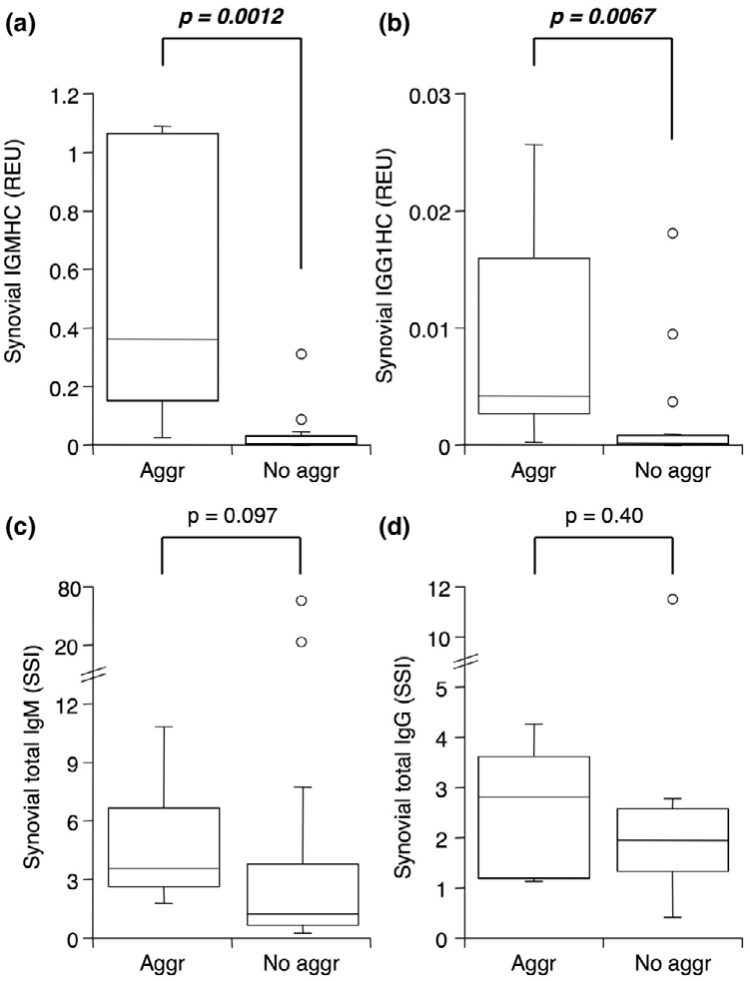
The presence of lymphoid aggregates in rheumatoid arthritis (RA) synovia is associated with elevated total immunoglobulin message, but not protein. GAPDH-normalized message for IgM **(a) **and IgG1 **(b) **heavy constant region in cDNA from RA synovia with (Aggr, n = 8) or without (No aggr, n = 17) lymphoid aggregates. Serum-normalized levels of **(c) **total IgM and **(d) **total IgG in extracts from the same synovia as in (a) and (b). Tissues were obtained by arthroplasty or arthroscopic shaver biopsy, and cDNA was analyzed by quantitative real-time polymerase chain reaction (a, b). Extracts were analyzed by enzyme-linked immunosorbent assay for antibodies of interest and albumin (c, d). See Figure 1 legend for box plot definitions. Indicated *P *values were determined by Wilcoxon rank sum test. GAPDH, glyceraldehyde-3-phosphate dehydrogenase; REU, relative expression units; SSI, synovial/serum index.

Lymphoid cell infiltrates in RA synovia can be more or less organized. In a subset of tissues, lymphoid cells are organized in aggregates that might function as ectopic lymphoid organs and contribute to local autoantibody production. To examine this possibility, the presence or absence of lymphoid aggregates in 25 RA synovial tissues was determined and SSI for RF-IgM and anti-CCP IgG calculated. Grade 2 or 3 lymphoid aggregates were identified in cryosections of eight synovia. As shown in Figure 4, both autoantibodies were present at significantly elevated levels in synovia containing lymphoid aggregates compared with synovia with diffuse lymphoid infiltration. In tissues with lymphoid aggregates, SSI for both autoantibodies was above 1 (*P *= 0.023 and 0.008, SSI above 1 in 7/8 and 8/8, respectively, for RF-IgM and anti-CCP IgG), whereas in tissues with diffuse infiltration the autoantibody SSI was not different from 1 (*P *= 0.60 and 0.19, SSI above 1 in 5/17 and 11/17, respectively). Total IgM and IgG were also determined in these extracts (Figure 5). Significantly enhanced IgM and IgG1 constant region message was detected by qPCR in synovia with lymphoid aggregates (Figure 5a,b). In contrast, the specific activities for IgM and IgG were similar in tissues with and without organized lymphoid infiltration (Figure 5c,d).

### Effect of rituximab on synovial autoantibody and immunoglobulin content

**Figure 6:**
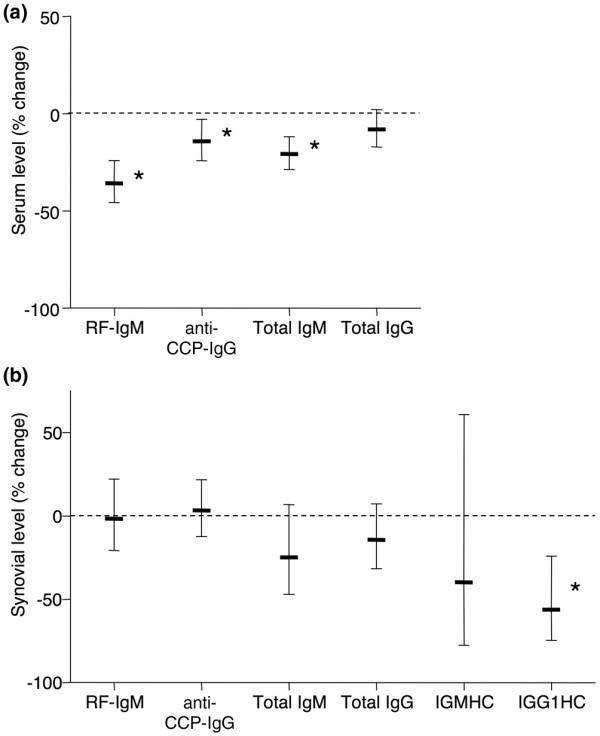
Effect of rituximab on circulating and synovial autoantibodies and immunoglobulin. **(a) **Circulating levels of autoantibodies and total IgM and IgG 8 weeks after rituximab treatment are expressed as percentage of pretreatment levels. **(b) **Serum-normalized levels of autoantibodies and total IgM and IgG determined by enzyme-linked immunosorbent assay, or mRNA levels of IgM and IgG1 heavy constant region determined by quantitative real-time polymerase chain reaction, in synovial biopsies 8 weeks after rituximab treatment are expressed as percentage of pretreatment levels. Data are expressed as geometric mean ± 95% confidence interval (CI) of 14 subjects. Asterisks denote that 95% CI excludes 0% change (stippled line). anti-CCP, anti-cyclic citrullinated peptide; RF-IgM, rheumatoid factor of the IgM subtype.

Baseline blood and synovial biopsies were collected prior to treatment with the B-cell-depleting antibody, rituximab. Eight weeks later, blood and synovial samples from the same joint were obtained. As described earlier, circulating B cells were nearly completely depleted by treatment (geometric mean depletion 98.8%, CI 97.7% to 99.3%). A small but significant reduction in circulating RF-IgM, anti-CCP IgG, and total IgM, but not total IgG, was observed (Figure 6a). However, the synovial content of autoantibodies and immunoglobulins did not change following rituximab treatment when all treated patients were considered as a single group (Figure 6b). There was, however, a significant reduction of IgG1 constant region message in synovial tissues after rituximab treatment (Figure 6b). The correlation between clinical response to rituximab, as measured by change in disease activity score using 28 joint counts (DAS28), and percentage change of SSI pre- to post-treatment was examined; however, none of the autoantibodies or total immunoglobulin examined covaried with DAS28 in a statistically significant manner.

### Differential effect of rituximab in synovial tissues containing lymphoid aggregates

**Figure 7:**
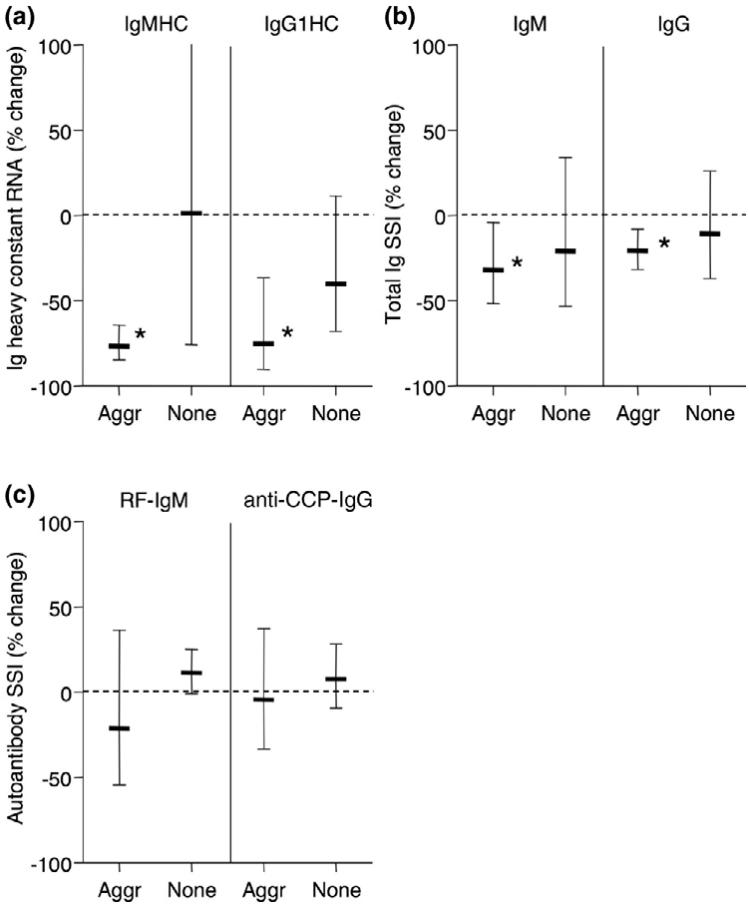
Rituximab selectively lowers total immunoglobulin synthesis but not autoantibody content in rheumatoid arthritis (RA) synovia containing lymphoid aggregates. **(a) **GAPDH-normalized synovial message for IgM and IgG1 heavy constant region in RA synovial biopsies with (Aggr, n = 5) or without (None, n = 9) lymphoid aggregates 8 weeks after rituximab treatment. Serum-normalized levels of **(b) **total IgM and total IgG and **(c) **RF-IgM and anti-CCP IgG in extracts from the same synovia as in (a). Data are expressed as geometric mean ± 95% confidence interval (CI) of post-treatment levels relative to pretreatment levels. Asterisks denote that 95% CI excludes 0% change (stippled line). anti-CCP, anti-cyclic citrullinated peptide; GAPDH, glyceraldehyde-3-phosphate dehydrogenase; RF-IgM, rheumatoid factor of the IgM subtype; SSI, synovial/serum index.

Trial subjects were grouped according to the presence or absence of lymphoid aggregates in their synovial biopsies prior to rituximab treatment, and the effect of rituximab was determined. Lymphoid aggregates were observed 8 weeks after rituximab treatment in all synovia that contained such aggregates prior to treatment (n = 5). The effect of rituximab on circulating autoantibodies or total immunoglobulins did not differ based on the presence of synovial aggregates. However, in synovia containing lymphoid aggregates, substantial and significant reductions of total IgM and IgG were noted in response to rituximab, whether assayed by constant region mRNA levels (Figure 7a) or by protein content (Figure 7b). Such decreases were not observed in synovial tissues lacking lymphoid aggregates (Figure 7). Despite the significant effect on total immunoglobulin content in aggregate-containing synovia, neither RF-IgM nor anti-CCP IgG in synovial tissues were decreased by rituximab, whether they contained lymphoid aggregates or not (Figure 7c).

## Discussion

Rituximab is a B-cell-depleting antibody approved for treatment of anti-tumor necrosis factor (TNF)-resistant RA but its mechanism of action is unclear. In the synovium, B cells and immunoglobulin constant region mRNA are significantly lowered in patients with a substantial (at or above American College of Rheumatology 50%) clinical response to rituximab [6]. The effect of rituximab on synovial autoantibody synthesis has not been previously reported, although circulating autoantibodies are known to be only modestly affected [5]. This paper describes the effect of rituximab on synovial autoantibody and immunoglobulin levels, as determined by a novel approach for the antibody measurement, in synovial biopsies from patients with RA.

In cross-sectional feasibility studies, both RF and anti-CCP were easily detected in the majority of the RA synovial extracts. The variability among patients was very large (80- to 100-fold when expressed without regard to serum content). To account for variable levels in serum and also to normalize for differing amounts of serum in the tissues, an albumin-normalized SSI was formulated whereby a value above 1 by definition indicates synovial enrichment (that is, a higher level than would be expected if the entire amount in synovium was associated with its serum content). A similar approach was employed earlier in synovial fluid [21]. Naturally, enrichment does not directly indicate synovial synthesis and alternatively can reflect accumulation of antibodies or immunoglobulin in the synovial environment for a variety of other reasons. However, significant correlations between total immunoglobulin mRNA levels (as determined by qPCR) and SSI were observed, indicating that locally synthesized antibodies provide an important component of SSI. In addition, this correlation suggests that the sampling methods developed for other synovial protein [18] and mRNA [19] analysis are applicable to immunoglobulin measurements.

Using this method, both anti-CCP IgG and anti-tetanus IgG as well as total IgM and IgG were found to be significantly enriched in RA synovia, whereas the value for RF-IgM did not differ significantly from 1. The reason for the RF-IgM results is unclear; certainly, production of RFs by synovial tissue has been demonstrated earlier [9,10,13-15], but much larger amounts made in lymph nodes and spleen might mask the synovial contribution to blood levels. More interesting, however, is the synovial enrichment of anti-tetanus IgG, an antibody irrelevant to the pathology of RA but earlier shown to be present in RA synovia [22]. This observation supports the idea that the RA synovium provides a favorable environment for any antibody-producing cell. This notion stems from observations that fibroblast-like synoviocytes support *in vitro *survival and differentiation of B cells [23,24]. Also, the inflamed RA synovium releases CXCL12 (SDF-1), interleukin-6, TNF, BAFF (B-cell activating factor of TNF family), and APRIL (a proliferation-inducing ligand), all known to promote the accumulation and survival of long-lived plasma cells, which are normally found mainly in bone marrow [25].

Lymphocytic infiltrates in rheumatoid synovia can be diffuse or alternatively occur in more or less organized aggregates, sometimes resembling germinal centers [26-28]. The impact of this lymphoid organization on autoantibody production remains poorly understood, although an association between circulating RFs and the presence of a synovial germinal center reaction has been described [27,28] as well as dismissed [29]. Our findings demonstrate for the first time that RA synovia containing lymphoid aggregates have significantly larger amounts of RF-IgM and anti-CCP IgG, after normalizing for serum content. It should also be noted that aggregate-containing tissues had autoantibody SSI values well above 1, indicating local synthesis and/or accumulation, whereas autoantibody SSI in tissues with diffuse lymphoid infiltration did not significantly differ from 1, suggesting the absence of local production. In accordance with earlier findings [8], RA synovia with lymphoid aggregates also contained elevated immunoglobulin constant region mRNA but this did not translate into a significant effect on total IgG or total IgM protein.

Finally, we determined the effect of rituximab on synovial autoantibody and immunoglobulin levels in the longitudinal ARISE study (described in detail earlier [6]). Despite the considerable clinical improvement induced by rituximab treatment, as well as the almost complete depletion of circulating B cells [3,4], only modest, albeit significant, decreases in circulating RF and anti-CCP were observed [5] and levels of these autoantibodies were still highly elevated compared with normal controls. In the present study, similar results were obtained. Furthermore, when all patients were considered together, there was no significant effect of rituximab on synovial content of any of the autoantibodies or immunoglobulin studied, with the lone exception of IgG heavy constant region message 8 weeks following rituximab infusion.

Of note, however, when patients were segregated according to the presence or absence of lymphoid aggregates in their synovia, the results were very different. Rituximab significantly reduced immunoglobulin production at the mRNA level, as well as total IgM and IgG content in synovia containing lymphoid aggregates, but not in synovia where lymphoid infiltration was diffuse. In spite of this, however, the treatment did not have any effect on RF-IgM or anti-CCP IgG content in synovia with lymphoid aggregates, even though these tissues produced massively increased levels of autoantibodies compared with synovia with diffuse infiltration.

It is possible that the differential effect of rituximab on total immunoglobulin and autoantibody synovial production is due to a systematic change in the half-life of autoantibody-producing cells such that it may compensate for any effects of rituximab. More likely, however, and in light of the recent finding that the size and number of synovial lymphoid aggregates in RA are unaltered early after rituximab treatment [7], the aggregate milieu might provide a protective niche for those B cells and plasma cells that produce arthritis-associated autoantibodies. In contrast, total immunoglobulins (presumably including disease-irrelevant antibodies) might be synthesized by B cells and plasma cells that are more sensitive to rituximab treatment. The existence of protective niches for certain classes of B cells can be seen in a mouse model in which an anti-CD20 antibody ineffectively depleted peritoneal B cells, despite almost complete removal of circulating B cells [30]. Similarly, in macaques, rituximab depleted circulating and splenic B cells, whereas the effect on B cells in lymph nodes and bone marrow was variable [31]. We [6] and others [7] have shown that rituximab significantly reduces synovial B cells in RA patients, but the effect is highly variable and sometimes nonexistent, suggesting protection or replenishment of the synovial B-cell pool. Recently, a more complete depletion of synovial B cells by rituximab was demonstrated at a later time point following treatment [32]; however, this could be explained by an overall reduction of synovitis leading to a secondary effect on lymphocyte accumulation.

## Conclusion

We developed quantitative methods for the determination of synovial autoantibody and immunoglobulin enrichment in biopsy materials obtained in clinical trials. Immunoglobulin mRNA correlated with synovial protein levels, consistent with local immunoglobulin synthesis. Both anti-CCP and RF-IgM were significantly enriched in synovial tissues containing lymphoid aggregates. However, in an 8-week study, autoantibody production in RA synovia was not altered by rituximab, whether aggregates were present or not. In addition, aggregates survived treatment. Thus, synovial lymphoid architecture is coupled with immunoglobulin and autoantibody production, but the role of synovial ectopic lymphoneogenesis in the pathogenesis and treatment of RA remains uncertain. It is unclear whether these events are critical to pathogenesis or whether they merely constitute epiphenomena of chronic inflammation. Further studies in patients with earlier disease and using other agents will be required to elucidate the contribution of synovial antibody production and lymphoid architecture to RA pathogenesis.

## Abbreviations

anti-CCP: anti-cyclic citrullinated peptide; ARISE: Assessment of Rituximab's Immunomodulatory Synovial Effects; CI: confidence interval; DAS28: disease activity score using 28 joint counts; GAPDH: glyceraldehyde-3-phosphate dehydrogenase; OA: osteoarthritis; qPCR: quantitative real-time polymerase chain reaction; RA: rheumatoid arthritis; REU: relative expression units; RF: rheumatoid factor; RF-IgM: rheumatoid factor of the IgM subtype; SSI: synovial/serum index; TNF: tumor necrosis factor.

## Competing interests

AK and DLB received financial support from Genentech (South San Francisco, CA, USA) to conduct the ARISE study. The other authors declare that they have no competing interests.

## Authors' contributions

SR participated in the design of the study, developed and performed molecular analysis, performed the statistical analysis, and drafted the manuscript. NW and KK performed arthroscopic procedures and collected samples. NZ participated in the design of antibody quantitative assays. AK and DLB conceived of, designed, and coordinated the study. DLB participated in drafting and editing the manuscript and is responsible for this manuscript. All authors read and approved the final manuscript.
